# Development and validation of a model to predict ceiling of care in COVID-19 hospitalized patients

**DOI:** 10.1186/s12904-024-01490-8

**Published:** 2024-07-16

**Authors:** N Pallarès, H Inouzhe, S Straw, N Safdar, D Fernández, J Cortés, L Rodríguez, S Videla, I Barrio, KK Witte, J Carratalà, C Tebé, MetroSud, Gabriela Abelenda-Alonso, Alexander Rombauts, Isabel Oriol, Antonella F. Simonetti, Alejandro Rodríguez-Molinero, Elisenda Izquierdo, Vicens Díaz-Brito, Carlota Gudiol, Judit Aranda-Lobo, Marta Arroyo, Carlos Pérez-López, Montserrat Sanmartí, Encarna Moreno, Maria C. Alvarez, Ana Faura, Martha González, Paula Cruz, Mireia Colom, Andrea Perez, Laura Serrano, DIVINE study group, Mireia Besalú, Erik Cobo, Leire Garmendia, Guadalupe Gómez, Pilar Hereu, Klaus Langohr, Gemma Molist, Núria Pérez-Álvarez, Xavier Piulachs

**Affiliations:** 1 Biostatistics Support and Research Unit, Germans Trias I Pujol Research Institute and Hospital (IGTP), Campus Can RutiCarretera de Can RutiCamí de Les Escoles S/N, Barcelona, Badalona 08916 Spain; 2 Department of Basic Clinical Practice, School of Medicine and Health Sciences, University of Barcelona, Barcelona, Spain; 3 Basque Center for Applied Mathematics, BCAM, Bilbao, Spain; 4 Leeds Institute of Cardiovascular and Metabolic Medicine, University of Leeds, Leeds, UK; 5 Department of Internal Medicine, St James’s University Hospitals, Leeds Teaching Hospitals NHS Foundation Trust, Leeds, UK; 6 Department of Internal Medicine, Pennsylvania Hospital, University of Pennsylvania Health System, Philadelphia, USA; 7 Department of Statistics and Operations Research, Universitat Politècnica de, Catalunya/BarcelonaTech, Barcelona, Spain; 8 Institute of Mathematics of UPC - BarcelonaTech (IMTech), Barcelona, Spain; 9 Centro de Investigación Biomédica en Red de Salud Mental, Instituto de Salud Carlos III (CIBERSAM), Madrid, Spain; 10 Department of Clinical Pharmacology, Bellvitge University Hospital, Barcelona, Spain; 11 Department of Pathology and Experimental Therapeutics, School of Medicine and Health Sciences, University of Barcelona, Barcelona, Spain; 12 Department of Mathematics, University of the Basque Country UPV/EHU, Leioa, Spain; 13 Department of Infectious Diseases, Bellvitge University Hospital, Barcelona, Spain; 14 Bellvitge Biomedical Research Institute (IDIBELL), Barcelona, Spain; 15 Centro de Investigación en Red de Enfermedades Infecciosas (CIBERINFEC), Instituto de Salud Carlos III, Madrid, Spain; 16 Department of Clinical Sciences, School of Medicine and Health Sciences, University of Barcelona, Barcelona, Spain

**Keywords:** COVID-19, Therapeutic ceiling of care, Prediction model, Bootstrapping, Calibration, Discrimination

## Abstract

**Background:**

Therapeutic ceiling of care is the maximum level of care deemed appropiate to offer to a patient based on their clinical profile and therefore their potential to derive benefit, within the context of the availability of resources. To our knowledge, there are no models to predict ceiling of care decisions in COVID-19 patients or other acute illnesses. We aimed to develop and validate a clinical prediction model to predict ceiling of care decisions using information readily available at the point of hospital admission.

**Methods:**

We studied a cohort of adult COVID-19 patients who were hospitalized in 5 centres of Catalonia between 2020 and 2021. All patients had microbiologically proven SARS-CoV-2 infection at the time of hospitalization. Their therapeutic ceiling of care was assessed at hospital admission. Comorbidities collected at hospital admission, age and sex were considered as potential factors for predicting ceiling of care. A logistic regression model was used to predict the ceiling of care. The final model was validated internally and externally using a cohort obtained from the Leeds Teaching Hospitals NHS Trust. The TRIPOD Checklist for Prediction Model Development and Validation from the EQUATOR Network has been followed to report the model.

**Results:**

A total of 5813 patients were included in the development cohort, of whom 31.5% were assigned a ceiling of care at the point of hospital admission. A model including age, COVID-19 wave, chronic kidney disease, dementia, dyslipidaemia, heart failure, metastasis, peripheral vascular disease, chronic obstructive pulmonary disease, and stroke or transient ischaemic attack had excellent discrimination and calibration. Subgroup analysis by sex, age group, and relevant comorbidities showed excellent figures for calibration and discrimination. External validation on the Leeds Teaching Hospitals cohort also showed good performance.

**Conclusions:**

Ceiling of care can be predicted with great accuracy from a patient’s clinical information available at the point of hospital admission. Cohorts without information on ceiling of care could use our model to estimate the probability of ceiling of care. In future pandemics, during emergency situations or when dealing with frail patients, where time-sensitive decisions about the use of life-prolonging treatments are required, this model, combined with clinical expertise, could be valuable. However, future work is needed to evaluate the use of this prediction tool outside COVID-19.

**Supplementary Information:**

The online version contains supplementary material available at 10.1186/s12904-024-01490-8.

## Background

Therapeutic ceiling of care is the maximum level of care deemed appropiate to offer to a patient. There is no consensus on the criteria for reaching a decision regarding ceiling of care, rather, these decisions are made between patients, their next-of-kin and treating teams taking into account the potential to derive benefit from intensive treatments and the availability of resources. There are limited data on how these decisions are made, and for whom, however during the coronavirus disease 2019 (COVID-19) pandemic, due to the excess demand for critical care and the limited availability of clinical resources, ceiling of care decisions often become routine. Previously published data [1, 2], suggest patients hospitalized with COVID-19 who had a ceiling of care decision below full intensive care-based treatment were mainly older, had more comorbidities, and paradoxically less severe symptoms and markers of disease severity at baseline. The incidence of death, severe pneumonia, and complications (cardiac and respiratory events, renal impairment, mental state alteration and nosocomial infection) was higher in patients with a therapeutic ceiling of care [1, 2], which is therefore a relevant source of bias for analyses aiming to explore factors associated with outcomes in this and other settings. Nevertheless, the number of studies on COVID-19 with information on patient’s ceiling of care is very limited, and mainly focused on specific treatments such as continuous positive airway pressure (CPAP) [3, 4] or non-invasive mechanical ventilation (NIMV) [5].

The lack of scientific evidence and medical consensus could lead to unwarranted variations in healthcare delivery [6], and the ceiling of care assignment is no exception. In this regard, the Australian recommendations for facilitating advanced care planning in the context of COVID-19 [7] are a step in the right direction. The same could be said about other initiatives to define an advance care plan for people with a complex chronic condition or who are likely to be nearing the end of life [8, 9]. However, in an unprecedented situation such as the COVID pandemic, decisions need to be made within hours, with little time to reach a consensus with the patients or their relatives. In this scenario, the availability of a reliable and easy to calculate clinical prediction tool to guide and support clinicians in their decision making, could be of great relevance. Ideally, the tool should correctly identify those patients who are unlikely to benefit from receiving intensive care. Moreover, when planning responses to future pandemics, the ability to predict for whom full intensive care-based treatments would be appropriate could help plan the allocation of resources.

The aim of this study was to develop and validate a model to predict the ceiling of care for hospitalized subjects with COVID-19 using information on the patient’s demographic and clinical profile available at the time of hospital admission.

## Methods

### Data source

The MetroSud study is an observational multicenter study conducted in five centres located in the south metropolitan area of Barcelona (Catalonia, Spain) [10]. The population of the MetroSud cohort has been described previously [1]. Briefly, it was a prospective cohort of consecutive adult patients with a proven SARS-CoV-2 infection admitted to 5 centres belonging to the southern metropolitan area of Barcelona in four waves of the pandemic. The first wave included hospitalized patients between March 1^st^ and April 15^th^, 2020; second wave, from October 1st to November 31^st^, 2020; third, from January 1st to February 28^th^, 2021; and fourth, from July 1st to August 31^st^, 2021 [11]. The first wave had a severe impact on the elderly, and the Spanish government imposed a national lockdown to reduce infection rates and reduce the burden on the healthcare system. The predominant variant in this first wave was the original SARS-CoV-2 strain, which was superseded by the alpha variant in waves 2 and 3. The vaccination campaign started at the end of December 2020, just before the inclusion of the third wave cases. The fourth wave, predominantly the Delta variant, mainly affected young people, who had not yet been vaccinated.

An electronic case report form in REDCap databases [12, 13] was designed ad hoc in March 2020 to collect study data. Demographic data, comorbidities and relevant findings on medical history, previous medications, vital signs and laboratory results were collected at baseline. Ceiling of care definition was agreed between participating centres and determined at hospital admission by the treating teams following discussion with patients and their next-of-kin. Patients without a ceiling of care would have access to intensive care unit (ICU) or would be able to receive invasive mechanical ventilation (IMV). Otherwise, patients assigned to ceiling of care would have limited access to the ICU and, if they require any respiratory support, it would be non-rebreather mask, high-flow nasal cannula or NIMV. The study was approved by the Bellvitge Hospital Research Ethics Committee with medicines (CREm) in accordance with Spanish legislation and was performed in accordance with the Helsinki Declaration of 1964. The need for patient informed consent was waived by the ethics committee. Bellvitge's CREm decision was the basis for the approval of the remaining hospital centres.

The Leeds Teaching Hospitals cohort [2] was used for external validation. Data comes from a retrospective observational study, where all consecutive patients aged ≥ 18 years with laboratory confirmed SARS-CoV-2 infection admitted to Leeds Teaching Hospitals between 5th March and 7th May 2020 were included. The inclusion period was within the first wave in England and the predominant strain of SARS-CoV2 was the original one. Thus, epidemic conditions and knowledge were similar, making the observation periods of both cohorts comparable, albeit within different healthcare and cultural settings. For this cohort, clinical data and outcomes were obtained from the Leeds Patient Pathway Manager Plus electronic care record. All patients were followed-up until hospital discharge or death. Ceiling of care decisions were standardised and electronically documented using the ReSPECT process [14].

### Statistical analysis

Logistic regression models were used to derive the predictive capability. First, a descriptive analysis of potential prognostic factors for ceiling of care was performed. The set of prognostic factors was agreed with the whole research team. These potential predictors were age, sex and main comorbidities at hospital admission such as: diabetes mellitus, chronic obstructive pulmonary disease (COPD), chronic renal disease, myocardial infarction, neoplasm (in three categories: no neoplasm, metastatic neoplasm, and non-metastatic neoplasm), heart failures, stroke or transient ischaemic attack (TIA), dementia, peripheral vascular disease, severe liver disease connective tissue disease, ulcer, mild liver disease, human immunodeficiency virus (HIV), hemiplegia, hypertension, and dyslipidemia. A dichotomous variable indicating whether the patient belonged to the first wave or not was also included, as the singularity of the circumstances that accompanied the first wave was not repeated in subsequent waves. Following this, 1000 bootstrap samples were generated [15, 16]. A logistic regression model with ceiling/no ceiling as outcome was fitted for each bootstrap sample using backward elimination. Variables that were selected in more than 95% of these 1000 models were candidates for the final model. Alternative variable selection methods such as Lasso regression, classification trees, and random forests were also applied to compare their results and, thereby increase the robustness of the selected variables (Supplementary File 3: Supplementary Table 1). The non-linear relationship between age and the log odd of the outcome was assessed. The model using a 2-grade age polynomial presented better likelihood, measured by the Akaike Information Criterion (AIC) [17], than the model using age as a linear term or a 3-grade age polynomial (Supplementary File 3: Supplementary Fig. 1 and Supplementary Table 2). Main interactions between selected variables were also assessed. Multicollinearity was analyzed using the variance inflation factor (VIF). Thereafter, the final set of included variables in the model was approved by the clinicians.

Internal validation of the resulting modelwas based on discrimination, calibration, and bootstrap validation using the development cohort [18]. Discrimination was assessed by estimating the area under the receiver operating characteristic curve (AUC) and the Brier score [19]. Calibration was assessed by graphically comparing the observed versus expected probabilities of ceiling of care by deciles of predicted risk. Bootstrap validation as recommended in the TRIPOD Statement [20] was also performed. This validation accounts for model overfitting and corrects optimism in the final prediction model, evaluating the possible overestimation of performance of the model on the development data. Internal validation was performed in the whole cohort and in pre-specified subgroups of clinical interest.

To our knowledge, there are no published data describing a cut-off for ceiling of care decision. Therefore, the percentage of patients correctly classified was calculated using the prevalence of ceiling of care in the development cohort (31.5%) as a threshold. Patients with a ceiling of care and a probability predicted by the model greater than 0.315, according to prevalence of ceiling of care, and patients with no ceiling of care assigned at hospital admission and a probability predicted by the model less than 0.315 were considered as correctly classified patients.

External validation was performed using a dataset from the Leeds Teaching Hospitals. Model discrimination was assessed by estimating the AUC and the Brier score, and model calibration was assessed by comparing observed versus expected probabilities of ceiling of care by deciles of predicted risk. Model validation was performed in the whole cohort and in the same subgroups as the internal one. The percentage of correctly classified patients in the external cohort was also calculated using the same cut-off threshold.

The MetroSud cohort included all consecutive patients as described above, and no formal sample size was computed in advance because of the exceptional circumstances of the pandemic. The TRIPOD Checklist for Prediction Model Development and Validation [20, 21] from the EQUATOR Network has been followed to report the development and validation of our model (Supplementary file 1). All analyses were performed with 95% confidence intervals and conducted using R software version 4.1.0 [22]. The main R packages used for data management and analysis were dplyr [23], REDCapDM [24], compareGroups [25], glmnet [26], bootStepAIC [27], rpart [28], rpart.plot [29] and Boruta [30].

## Results

**Fig. 1:**
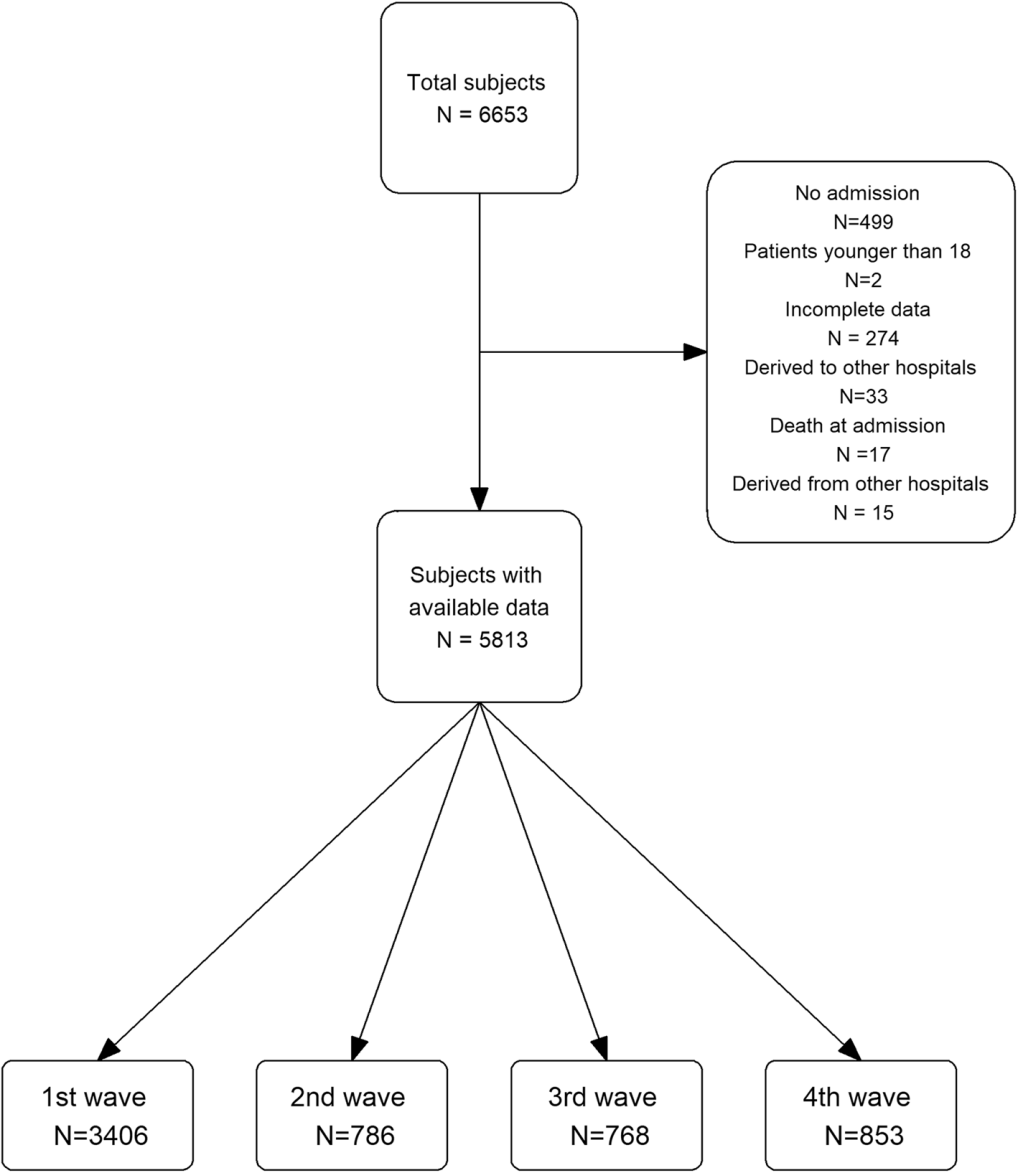
Flow chart of patients

The number of patients included in the cohort as well the number used to develop the model are shown in Fig. 1 (Flowchart). A total of 6653 individuals were included in the MetroSud cohort. Patients who were admitted to the hospital for less than 24 h (*N* = 499), who died within the first 24 h (*N* = 17), who had missing values in a pool of essential variables (age, sex, comorbidities needed to compute the Charlson score, ceiling of care, and circumstances at discharge) (*N* = 274), or those patients admitted firstly in one of the Metrosud five centres but transferred for treatment to another (*N* = 48) were excluded from the analysis, leaving a total of 5813 individuals for model development.

After the performed selection process, the variables that were consistently retained in over 95% of the estimated models included age, chronic kidney disease, dementia, heart failure, neoplasm (in three categories: no neoplasm, neoplasm without metastasis, and neoplasm with metastasis), wave (in two categories: 1st wave vs other), peripherical vascular disease, COPD, and stroke or transient ischaemic attack. Initially excluded factors were reviewed by the clinicians in the study group. For instance, hypertension was initially excluded because it was retained in only 19% of the models, but was eventually included in the final model because clinicians considered it relevant to the assignment of ceiling of care. The main interactions between variables were assessed and only the interaction between age and dementia was included in the final model due to its clinical relevance and an improvement in the AIC estimator [31]. In the multicollinearity analysis between variables in the final model, all variables presented a VIF < 4. The odds ratio (OR) and 95% CI for the final logistic model are presented in Table 1. The model equation and an illustrative example for the calculation of the ceiling of care probability are reported in Supplementary File 2.

**Table 1:** Odds ratio (OR), 95% confidence intervals (CI), and *p*-values for ceiling of care predictors in the multivariable logistic model

Predictors	Odds ratios	95% CI	*p*-values
(Intercept)	7.376	2.035 – 24.773	0.002
Age	0.803	0.771 – 0.837	< 0.001
Age^2^	1.003	1.002 – 1.003	< 0.001
Chronic kidney disease	2.040	1.451 – 2.886	< 0.001
Hypertension	0.950	0.799 – 1.128	0.559
Heart failure	1.933	1.448 – 2.592	< 0.001
Neoplasm without metastasis	1.829	1.403 – 2.388	< 0.001
Neoplasm with metastasis	6.158	3.251 – 11.978	< 0.001
Wave 2–3-4 (vs 1)	0.247	0.208 – 0.293	< 0.001
Peripherical vascular disease	1.992	1.437 – 2.771	< 0.001
COPD	1.484	1.234 – 1.785	< 0.001
Stroke or transient ischaemic attack	1.500	1.122 – 2.011	0.006
Age x dementia	1.183	1.141 – 1.227	< 0.001
Age^2^ x dementia	0.998	0.998 – 0.998	< 0.001

**Fig. 2:**
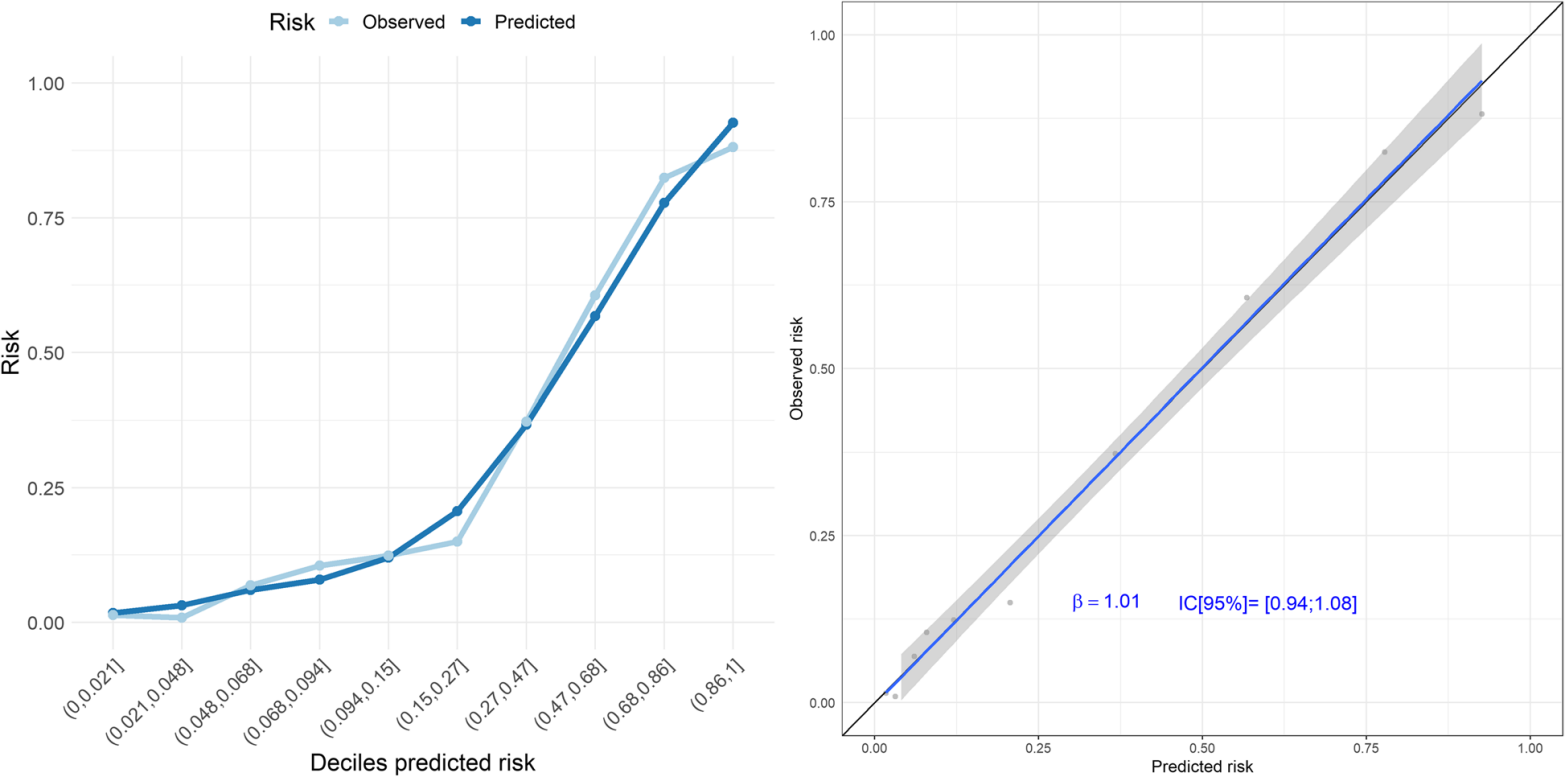
Observed vs predicted risk of the ceiling of care in the development cohort. Calibration Plot (left). The predicted event rate for each decile of predicted risk is the average of the probabilities of the patients in the decile (dark blue line). The observed event rate is the number of patients with a ceiling of care in the decile divided by the number of patients in the decile (light blue line). Ideally, the expected and observed rate lines should be overlapped. Calibration Plot (right): The calibration curve shows the relationship between the predicted and observed ceiling of care risk by deciles of predicted risk. The diagonal line represents the performance of an ideal model. The blue line represents the actual model performance comparing the predicted and observed risk. Points below the diagonal line correspond to over-prediction, whereas points above the diagonal line correspond to under-prediction

In the development cohort, the model yielded an AUC of 0.898 (95% CI 0.889 to 0.907) and a Brier score of 0.113. Calibration plots of observed versus predicted ceiling of care in deciles of predicted risk showed accuracy and the slope of the regression line between observed and predicted ceiling of care was close to 1 (95% CI 0.94 to 1.08) (Fig. 2). Using the prevalence of the ceiling of care in the development cohort (31.5%) as a cut-off, 83.38% of the patients were correctly classified by the model. Regarding the bootstrap validation as recommended in TRIPOD, 100 bootstrap samples were generated, and the best prediction model was estimated in each sample. When comparing the AIC and the slope of the regression line of these models in the bootstrap sample and in the original sample, the differences in both performance measures followed normal distributions of means and standard deviations close to 0.

To evaluate model performance, we conducted subgroup analyses based on clinical factors of interest, including age by deciles, sex, diabetes mellitus type 2, COPD, and hypertension. The results showed excellent calibration and discrimination measures, indicating a very reliable performance of the model (Supplementary File 4). Regarding deciles of age, the model performed better for patients younger than 65 years and older than 80 years than for patients between 65 and 80 years: more than 80% of patients younger than 65 years or older than 80 years were correctly classified, whereas for deciles of age (66, 71], (71, 75] and (75, 80] the percentages were 75.65%, 61.31% and 67.25% respectively.

Some differences were observed between the development and the external validation cohorts. Mainly, patients in the Leeds cohort were, in higher proportion, women, older, had a higher prevalence of most of the comorbidities at hospital admission, and almost 70% of patients had a ceiling of care assigned at hospital admission (compared with only 31.5% in the development cohort). In addition, the patients in the Leeds cohort were all from the first wave: if the comparison is restricted to 1^st^ wave patients in the development cohort, the differences slightly reduced (Table 2).

**Table 2:** Baseline patient’s characteristics of Catalan cohort (all waves), Catalan cohort (1st wave) and Leeds cohort

	Development cohort	Development cohort (1st wave)	Leeds cohort
	*N* = 5813	*N* = 3406	*N* = 404
Sex, N (%):			
Woman	2363 (40.7%)	1420 (41.7%)	220 (54.5%)
Age, Median [Q1;Q3]	66.0 [53.0;77.0]	68.0 [54.0;78.0]	77.0 [62.8;85.0]
Diabetes mellitus, N (%)	1352 (23.3%)	832 (24.4%)	124 (30.7%)
COPD, N (%)	1099 (18.9%)	599 (17.6%)	62 (15.3%)
Chronic kidney disease, N (%)	287 (4.94%)	198 (5.81%)	103 (25.5%)
Heart failure, N (%)	418 (7.19%)	244 (7.16%)	66 (16.3%)
Stroke or transient ischaemic attack, N (%)	366 (6.30%)	230 (6.75%)	48 (11.9%)
Peripheral vascular disease, N (%)	267 (4.59%)	137 (4.02%)	14 (3.47%)
Dementia, N (%)	397 (6.83%)	247 (7.25%)	85 (21.0%)
Hypertension, N (%)	2803 (48.2%)	1673 (49.1%)	186 (46.0%)
Neoplasm, N (%):			
No neoplasm	5355 (92.1%)	3151 (92.5%)	369 (91.3%)
Neoplasm without metastasis	398 (6.85%)	223 (6.55%)	25 (6.19%)
Neoplasm with metastasis	60 (1.03%)	32 (0.94%)	10 (2.48%)
Ceiling of care, N (%)	1831 (31.5%)	1330 (39.0%)	278 (68.8%)

**Fig. 3:**
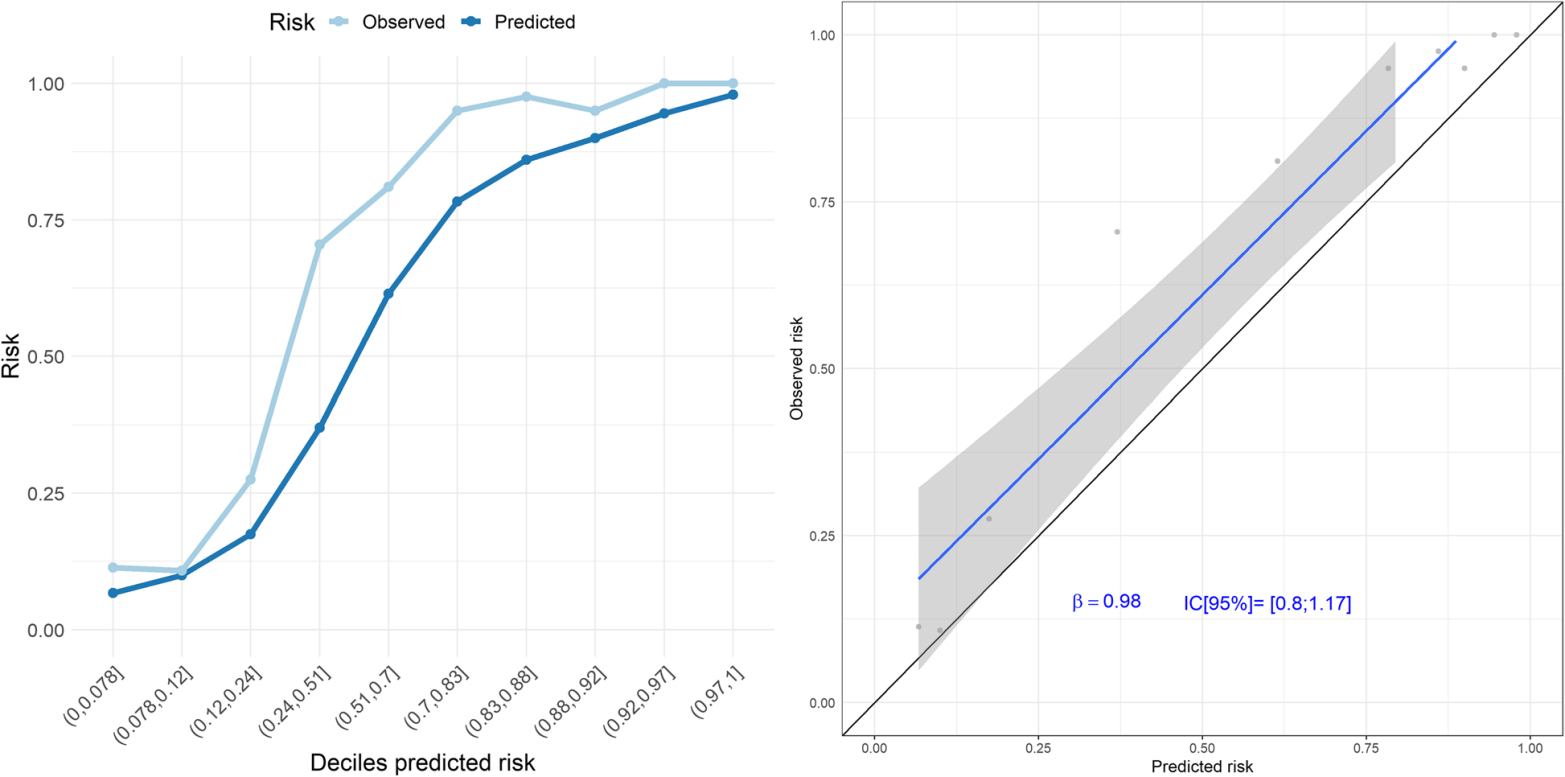
Observed vs predicted risk of the ceiling of care in the external cohort. Calibration Plot (left). The predicted event rate for each decile of predicted risk is the average of the probabilities of the patients in the decile (dark blue line). The observed event rate is the number of patients with a ceiling of care in the decile divided by the number of patients in the decile (light blue line). Ideally, the expected and observed rate lines should be overlapped. Calibration Plot (right): The calibration curve shows the relationship between the predicted and observed ceiling of care risk by deciles of predicted risk. The diagonal line represents the performance of an ideal model. The blue line represents the actual model performance comparing the predicted and observed risk. Points below the diagonal line correspond to over-prediction, whereas points above the diagonal line correspond to under-prediction

External validation of the ceiling of care model in the Leeds Teaching Hospitals cohort showed an AUC of 0.934 (95% CI 0.908 to 0.959) and a Brier score of 0.110. In terms of calibration, the model slightly underestimated the risk of ceiling of care in most of the deciles and the regression line appears slightly above the diagonal (Fig. 3). The percentage of patients correctly classified (using the MetroSud prevalence as threshold) in the external cohort was 87.87%. Validation in the Leeds subgroups showed similar results to the development cohort. The percentage of correctly classified patients in under 65 years and over 80 years patients was higher than 80% and was around 70% for those aged 65–79 years.

## Discussion

In this study we developed and validated a model to predict ceiling of care decisions based on clinical and demographic data readily available at the point of hospital admission for patients with COVID-19. The model showed good accuracy in both the internal and external validation cohorts. These patient factors were broadly in line with known predictors of poor outcomes within the context of COVID-19 and other acute illnesses, particularly more advanced age, dementia and major comorbidities.

All of the factors identified and ultimately selected for our model have previously been recognized as factors to be considered when assessing life-prolonging treatments and interventions [32–34]. Savulescu et al*.* [35] proposed an algorithm for rationing life-sustaining treatment during the COVID-19 pandemic, which included comorbidities and age as key factors when determining which patients have potential to derive benefit.

In our analysis, we explored the linearity of the ceiling of care by age (Supplementary File 3: Supplementary Fig. 1 and Supplementary Table 2). When age was treated as a non-linear factor, i.e., the effect of age on the ceiling of care was not constant, the model performance increased substantially. Therefore, we considered different slopes on risk increase per year, with a soft increase until approximately 65 years old and a sharp increase for older people. Age is an established risk factor for adverse outcomes for patients hospitalized with COVID-19, and in view of this, it seems to have often been taken into consideration when initiating a ceiling of care decision below full intensive care-based treatments.

To the best of our knowledge, this is the first model developed to predict the ceiling of care for COVID-19 patients. According to a live systematic review, current COVID-19 prediction models focus on predicting diagnostic, mortality, progression to severe disease, ICU admission and mechanical ventilation, intubation or length of hospital stay prediction [36]. However, most of the developed models were at high risk of bias and poorly reported [37], and had common issues previously identified [38]. The most frequent of those issues was patient selection bias. The main reasons identified were developing the model using participants that may not be representative of the model’s target population, and unclear reporting of included participants. In our model, we have taken these issues into account and we have reported them according to the TRIPOD guidelines.

Studies that have attempted to explore prognostic factors in COVID-19 have usually not accounted for the confounding effects of ceiling of care decisions. It is known that the incidence of death, severe pneumonia, and complications is higher in patients with a ceiling of care [1, 2]. Therefore, analysis of hospitalized subjects with SARS-CoV-2 infection should be stratified by ceiling of care to avoid bias and overestimation of outcomes in patients without ceiling of care. Thus, all these models could take advantage of our model and use it to stratify patients by ceiling of care. In addition, COVID-19 cohorts lacking information on the ceiling of care could use our proposed model to predict the ceiling of care for each patient, enabling accurate reporting of outomes according to the ceiling of care.

Potentially, our model could also be used in future pandemics or in frail patients to predict the ceiling of care of patients at hospital admission. It would therefore help to identify a proportion of patients for whom the intensive care-based treatments would not be appropriate, or on the other hand importantly determine for which patients full intensive care-based treatments might be appropriate so that resources can be effectively allocated. All of the variables in our model have previously been recognised as factors to be considered in the assessment of life-prolonging treatments and interventions, as they are routinely collected at hospital admission. Only the wave variable (i.e., first COVID-19 pandemic wave vs other) is a specific COVID-19 variable. Nonetheless, this variable acts as a proxy of the burden of care that hospitals experienced during the COVID-19 pandemic. Applying our model in a non-COVID situation would allow this variable to be used to identify scenarios with limited availability of resources (ICU beds, number of non-invasive ventilators or number of high-flow nasal oxygen therapy devices). However, the application of our model to other scenarios would require specific validation. Although a final decision to limit life-prolonging treatments should always be based on the patients’ advanced care plan and clinical judgement [39, 40], our model’s probability estimation of the ceiling of care could be presented to clinicians to add information to this discussion.

This study has several strengths, including a large sample size, the utilization of data from four different waves of the pandemic, robust statistical methods, and both internal and external validation of the developed model. To best of our knowledge, this is the first description of how ceiling of care decisions are made across different healthcare systems, in different countries, with very different provision of ICU beds, different ways in which healthcare is funded, and the culture/attitudes of patients. The main limitation of our study is that the data used to develop the model come from a metropolitan area in Catalonia (Spain), and generalizability of our findings may be limited due to differences between countries and between hospital resources available at different COVID-19 points in time [41]. Nevertheless, external validation in the Leeds Teaching Hospitals cohort showed comparable performance to the development cohort, suggesting potential applicability beyond the original cohort. Another limitation is that our outcome variable is not something that can be measured, but something that is assigned by the attending physician according to its judgement, which could lead to an observer bias [42]. Nevertheless, our definition was consistent with those used in the Leeds cohort [2], as well as a similar one used in an additional study to determine the factors that influence ceiling of treatment in an Emergency Department [32]. Clinical guidelines to assess the ceiling of care could help to add consensus to clinical judgement and reduce this bias. Moreover, the use of this model for patient's clinical management is not recommended until approval from regulatory authorities.

Although personalized prediction for each patient is the main goal, even when working with large datasets, the model we obtained estimates the probability of receiving a ceiling of care for patients with a certain profile. The probability obtained refers to the number of individuals who are expected to receive a ceiling of care given 100 individuals with the same profile, not the probability of ceiling of care for a specific individual. Patients may have a non-measured risk factor by our model that could modify its individual risk. We try to minimize the impact of these non-measured risk factors by using large datasets and then checking that the model performs well internally and externally.

## Conclusions

We have developed a model to predict the ceiling of care based on data readily available at the point of hospital admission for patients with COVID-19. The model showed good accuracy in the internal and external validation cohort and can be used to predict the ceiling of care in COVID-19 cohorts already collected. Potentially, the model could also be used in other scenarios where decisions about the ceiling of care are required. Further research is needed to apply this prediction tool in other settings and in future pandemics.

### Supplementary Information

Supplementary Material 1.

Supplementary Material 2.

Supplementary Material 3.

Supplementary Material 4.

## Data Availability

The datasets used and/or analysed during the current study are available from the corresponding author on reasonable request.

## Abbreviations

AIC: Akaike information criterion
AUC: Area under the receiver operating characteristic curve
CI: Confidence interval
COPD: Chronic obstructive pulmonary disease
CPAP: Continuous positive airway pressure
HIV: Human immunodeficiency virus
ICU: Intensive care unit
IMV: Invasive mechanical ventilation
NIMV: Non-invasive mechanical ventilation
TIA: Transient ischemick attack
ReSPECT: Recommended summary plan for emergency care and rreatment
OR: Odds ratio
VIF: Variance inflation factor

## References

[1] Pallarès N, Tebé C, Abelenda-Alonso G, Rombauts A, Oriol I, Simonetti AF (2023). Characteristics and outcomes by ceiling of care of subjects hospitalized with COVID-19 during four waves of the pandemic in a metropolitan area: a multicenter cohort study. Infect Dis Ther.

[2] Straw S, McGinlay M, Drozd M, Slater TA, Cowley A, Kamalathasan S (2021). Advanced care planning during the COVID-19 pandemic: ceiling of care decisions and their implications for observational data. BMC Palliat Care.

[3] Bradley P, Nixon J, Wilson J, Redfern J, Saba T, Nuttall E (2021). Continuous positive airway pressure (CPAP) as a ceiling of care treatment for hypoxaemic respiratory failure due to COVID-19. Clin Med Lond Engl.

[4] Walker J, Dolly S, Ng L, Prior-Ong M, Sabapathy K (2020). The role of CPAP as a potential bridge to invasive ventilation and as a ceiling-of-care for patients hospitalized with Covid-19-an observational study. PLoS One.

[5] Ramirez GA, Bozzolo EP, Gobbi A, Castelli E, Centurioni C, DI Meo M (2022). Outcomes of noninvasive ventilation as the ceiling of treatment in patients with COVID-19. Panminerva Med.

[6] Wennberg JE (1984). Dealing with medical practice variations: a proposal for action. Health Aff Proj Hope.

[7] Sinclair C, Nolte L, White BP, Detering KM (2020). Advance care planning in Australia during the COVID-19 outbreak: now more important than ever. Intern Med J..

[8] Medicina Paliativa. Available from: https://www.medicinapaliativa.es/La-planificacion-compartida-de-la-atencion-en-personas-con-enfermedad-oncologica-en-un-instituto-monografico-de-cancer-estudio-descriptivo-retrospectivo674. Cited 2023 May 15.

[9] Rietjens JAC, Sudore RL, Connolly M, van Delden JJ, Drickamer MA, Droger M (2017). Definition and recommendations for advance care planning: an international consensus supported by the European Association for Palliative Care. Lancet Oncol.

[10] Regió Sanitària Barcelona. CatSalut. Servei Català de la Salut. Available from: https://catsalut.gencat.cat/ca/coneix-catsalut/catsalut-territori/barcelona/. Cited 2023 Mar 26.

[11] Dades COVID. Available from: https://dadescovid.cat. Cited 2024 May 13.

[12] Harris PA, Taylor R, Thielke R, Payne J, Gonzalez N, Conde JG (2009). Research electronic data capture (REDCap)–a metadata-driven methodology and workflow process for providing translational research informatics support. J Biomed Inform.

[13] Harris PA, Taylor R, Minor BL, Elliott V, Fernandez M, O’Neal L (2019). The REDCap consortium: Building an international community of software platform partners. J Biomed Inform.

[14] Resuscitation Council UK. ReSPECT for healthcare professionals. Available from: https://www.resus.org.uk/respect/respect-healthcare-professionals. Cited 2023 Mar 26.

[15] Austin PC, Tu JV. Bootstrap Methods for Developing Predictive Models. Am Stat. 2012. Available from: https://www.tandfonline.com/doi/abs/10.1198/0003130043277. Cited 2023 Jul 13.

[16] Bootstrap SJ, Selection M (1996). Bootstrap model selection. J Am Stat Assoc.

[17] Akaike H (1974). A new look at the statistical model identification. IEEE Trans Autom Control.

[18] Steyerberg EW, Harrell FE, Borsboom GJ, Eijkemans MJ, Vergouwe Y, Habbema JD (2001). Internal validation of predictive models: efficiency of some procedures for logistic regression analysis. J Clin Epidemiol.

[19] Steyerberg EW, Vickers AJ, Cook NR, Gerds T, Gonen M, Obuchowski N (2010). Assessing the performance of prediction models: a framework for traditional and novel measures. Epidemiol Camb Mass.

[20] Moons KGM, Altman DG, Reitsma JB, Ioannidis JPA, Macaskill P, Steyerberg EW (2015). Transparent Reporting of a multivariable prediction model for Individual Prognosis or Diagnosis (TRIPOD): explanation and elaboration. Ann Intern Med.

[21] Collins GS, Reitsma JB, Altman DG, Moons KG (2015). Transparent reporting of a multivariable prediction model for individual prognosis or diagnosis (TRIPOD): the TRIPOD Statement. BMC Med.

[22] The Comprehensive R Archive Network. Available from: https://cran.r-project.org/. Cited 2023 Mar 19.

[23] Wickham H, François R, Henry L, Müller K, Vaughan D, Software P, et al. dplyr: A Grammar of Data Manipulation. 2023. Available from: https://CRAN.R-project.org/package=dplyr. Cited 2023 Apr 7.

[24] Carmezim J, Satorra P, Peñafiel J, García-Lerma E, Pallarès N, Santos N, Tebé C. REDCapDM: An R package with a set of data management tools for a REDCap project. BMC Med Res Methodol. 2024;24(1):55. 10.1186/s12874-024-02178-6 PMC10905808 38429658

[25] Subirana I, Salvador J. compareGroups: Descriptive Analysis by Groups. 2022. Available from: https://CRAN.R-project.org/package=compareGroups. Cited 2023 Apr 7.

[26] Friedman J, Hastie T, Tibshirani R, Narasimhan B, Tay K, Simon N, et al. glmnet: Lasso and Elastic-Net Regularized Generalized Linear Models. 2023. Available from: https://CRAN.R-project.org/package=glmnet. Cited 2023 Apr 7.

[27] Rizopoulos D. bootStepAIC: Bootstrap stepAIC. 2022. Available from: https://CRAN.R-project.org/package=bootStepAIC. Cited 2023 Apr 7.

[28] Therneau T, Atkinson B, port BR (producer of the initial R, maintainer 1999–2017). rpart: Recursive Partitioning and Regression Trees. 2022. Available from: https://cran.r-project.org/web/packages/rpart/index.html. Cited 2023 Jul 25.

[29] Milborrow S. rpart.plot: Plot ‘rpart’ Models: An Enhanced Version of ‘plot.rpart’. 2022. Available from: https://cran.r-project.org/web/packages/rpart.plot/index.html. Cited 2023 Jul 25.

[30] Kursa MB, Rudnicki WR (2010). Feature Selection with the Boruta Package. J Stat Softw.

[31] Bianchetti A, Rozzini R, Guerini F, Boffelli S, Ranieri P, Minelli G (2020). Clinical presentation of COVID19 in dementia patients. J Nutr Health Aging.

[32] Walzl N, Jameson J, Kinsella J, Lowe DJ (2019). Ceilings of treatment: a qualitative study in the emergency department. BMC Emerg Med.

[33] Rodríguez-Molinero A, López-Diéguez M, Tabuenca AI, de la Cruz JJ, Banegas JR (2010). Physicians’ impression on the elders’ functionality influences decision making for emergency care. Am J Emerg Med.

[34] Helsedirektoratet. 2023. Engelsk versjon. Available from: https://www.helsedirektoratet.no/veiledere/beslutningsprosesser-ved-begrensning-av-livsforlengende-behandling/engelsk-versjon. Cited 2023 Mar 26.

[35] Savulescu J, Vergano M, Craxì L, Wilkinson D (2020). An ethical algorithm for rationing life-sustaining treatment during the COVID-19 pandemic. BJA Br J Anaesth.

[36] Wynants L, Van Calster B, Collins GS, Riley RD, Heinze G, Schuit E (2020). Prediction models for diagnosis and prognosis of covid-19: systematic review and critical appraisal. BMJ.

[37] Collins GS (2022). COVID-19 prediction models need robust and transparent development. Disaster Med Public Health Prep.

[38] Steyerberg EW, Uno H, Ioannidis JPA, van Calster B, Ukaegbu C, Dhingra T (2018). Poor performance of clinical prediction models: the harm of commonly applied methods. J Clin Epidemiol.

[39] Herreros B, Gella P, de Asua Real D (2020). Triage during the COVID-19 epidemic in Spain: better and worse ethical arguments. J Med Ethics..

[40] Emanuel EJ, Persad G, Upshur R, Thome B, Parker M, Glickman A (2020). Fair allocation of scarce medical resources in the time of Covid-19. N Engl J Med.

[41] Rhodes A, Ferdinande P, Flaatten H, Guidet B, Metnitz PG, Moreno RP (2012). The variability of critical care bed numbers in Europe. Intensive Care Med.

[42] Hróbjartsson A, Thomsen ASS, Emanuelsson F, Tendal B, Hilden J, Boutron I (2012). Observer bias in randomised clinical trials with binary outcomes: systematic review of trials with both blinded and non-blinded outcome assessors. BMJ.

