# Supplementary material for: Development and validation of a model to predict ceiling of care in COVID-19 hospitalized patients
Source: BMC Palliat Care. 2024 Jul 16;23:173. doi: 10.1186/s12904-024-01490-8 (PMC11250965; doi:10.1186/s12904-024-01490-8)
Supplement: Supplementary file 2 — Supplementary Material 2. [file 12904_2024_1490_MOESM2_ESM.docx]

**Supplementary File 2**: Calculation of ceiling of care in a new COVID-19 hospitalized patient

To estimate the probability of receiving ceiling of care of a particular patient, one should calculate:

$P\left( ceiling of care \right)$= $\frac{e^{{(\beta}_{0}+\sum\beta x)}}{1+e^{{(\beta}_{0}+\sum\beta x)}}$

Where$\beta_{0}$ stands for the intercept of the model and $\sum\beta x$ stands for the linear function of key predictors in the model. Each predictor is multiplied by its corresponding $\beta$ as in the table below.

For illustrative purposes, we calculate here the ceiling of care prediction for a 75-year-old woman with dementia, hypertension, heart failure and COPD and belonging to the 2nd wave.

| Factor | $\beta$ | Patient | Result |
| --- | --- | --- | --- |
| (Intercept) | 1.9982280 | - | 1.9982280 |
| Age | -0.2194683 | 75 | -0.2194683*75= -16.46012 |
| Age^2^ | 0.0025310 | 75^2^ | 0.0025310*75^2^= 14.23687 |
| Chronic kidney disease | 0.7130276 | No | 0.7130276 *0=0 |
| Hypertension | -0.0513495 | Yes | -0.0513495*1= -0.0513495 |
| Heart failure | 0.6590353 | Yes | 0.6590353*1=0.6590353 |
| Neoplasm without metastasis | 0.6039457 | No neoplasm | 0.6039457*0= 0 |
| Neoplasm with metastasis | 1.8177804 | No neoplasm | 1.8177804*0= 0 |
| Wave 2-3-5 (vs 1) | -1.3974752 | Wave 2 | -1.3974752*1= -1.3974752 |
| Peripherical vascular disease | 0.6892895 | No | 0.6892895*0= 0 |
| COPD | 0.3949243 | Yes | 0.3949243*1= 0.3949243 |
| Stroke or transient ischaemic attack | 0.4051668 | No | 0.4051668*0= 0 |
| Age x dementia | 0.1682617 | 75, Yes | 0.1682617*75*1= 12.61963 |
| Age^2^ x dementia | -0.0019471 | 75^2^, Yes | -0.0019471*75^2^*1= -10.95244 |
| $\beta_{0}+\sum\beta x$ | | | 1.047303 |
| $\exp\left( \beta_{0}+\sum\beta x \right)$ | | | exp(1.047303)= 2.849954 |
| 1+$\exp\left( \beta_{0}+\sum\beta x \right)$ | | | 1+ 2.849954= 3.849954 |
| $\frac{\exp\left( \beta_{0}+\sum\beta x \right)}{1+exp\left( \beta_{0}+\sum\beta x \right)}$ | | | 2.849954/3.849954= 0.7402566 |
| $P\left( ceiling of care \right)$ | | | 74.03 |

Based on this, the risk of such a patient receiving ceiling of care is 74.03%.
