# Supplementary material for: Development and validation of a model to predict ceiling of care in COVID-19 hospitalized patients
Source: BMC Palliat Care. 2024 Jul 16;23:173. doi: 10.1186/s12904-024-01490-8 (PMC11250965; doi:10.1186/s12904-024-01490-8)
Supplement: Supplementary file 3 — Supplementary Material 3. [file 12904_2024_1490_MOESM3_ESM.docx]

SUPPLEMENTARY FILE

Supplementary Table 1. Variables selected in each of the variable selection methods used: bootstrapping, Lasso regression, classification trees and random forests. Variables selected to be included in the final model were variables that were selected in at least two of the four methods.

| **Variable** | **Bootstrapping** | **Lasso regression** | **Classification tree** | **Random forest** | **Total** |
| --- | --- | --- | --- | --- | --- |
| Age | Yes | Yes | Yes | Yes | 4 |
| Chronic kidney disease | Yes | Yes | Yes | Yes | 4 |
| Dementia | Yes | Yes | Yes | Yes | 4 |
| Heart failures | Yes | Yes | Yes | Yes | 4 |
| Wave | Yes | Yes | Yes | Yes | 4 |
| Stroke or transient ischaemic attack | Yes | Yes | Yes | Yes | 4 |
| Dyslipidemia | Yes | Yes | No | No | 2 |
| Neoplasm | Yes | Yes | No | No | 2 |
| Peripheral vascular disease | Yes | Yes | No | No | 2 |
| Chronic obstructive pulmonary disease | Yes | Yes | No | No | 2 |
| Hypertension | No | No | Yes | Yes | 2 |
| Myocardial infarction | No | Yes | No | No | 1 |
| Human immunodeficiency virus | No | No | No | No | 0 |
| Mild liver disease | No | No | No | No | 0 |
| Hemiplegia | No | No | No | No | 0 |
| Diabetes mellitus | No | No | No | No | 0 |
| Sex | No | No | No | No | 0 |
| Connective tissue disease | No | No | No | No | 0 |
| Ulcer | No | No | No | No | 0 |
| Severe liver disease | No | No | No | No | 0 |

Supplementary Figure 1. Relationship between age (in years) and ceiling of care. The effect of age on ceiling of care is not constant: we should consider different slopes on risk increase per year, with a soft increase until approximately 65 years old and a sharp increase for older people.


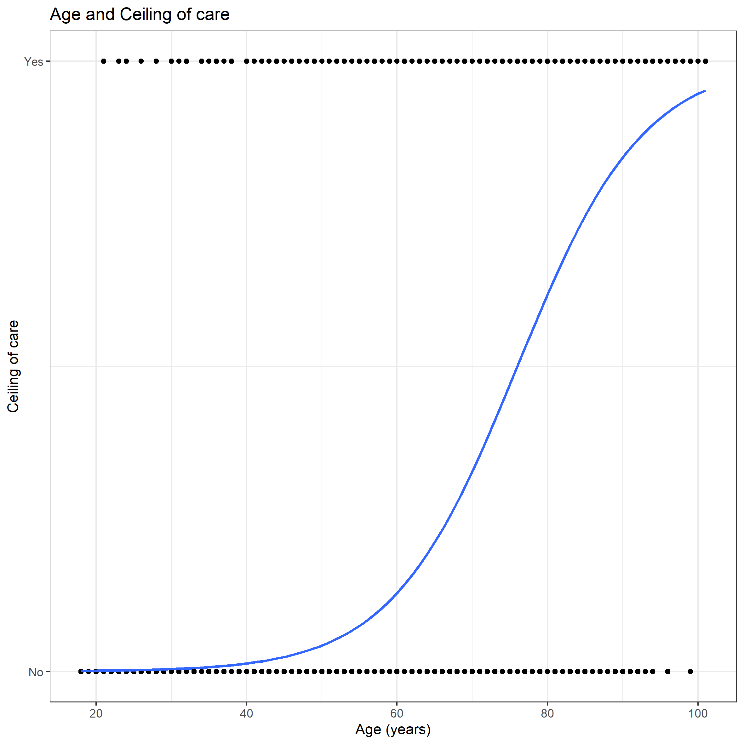


Supplementary Table 2. Comparison of all considered models regarding the relationship between age and ceiling of care. Akaike information criterion (AIC) for models considered age in four different ways: linear term, linear and quadratic term, age in a quadratic spline, and age in a cubic spline. P-value obtained from Likelihood Ratio Tests (LRT) comparing models.

| **Relationship between age and ceiling of care** | **AIC** | **p-value from a LRT** |
| --- | --- | --- |
| M1: Linear term of age | 4527.192 |  |
| M2: Linear and quadratic term of age | 4383.626 | M1 vs M2: p-value < 0.001 |
| M3: Quadratic spline for age | 4383.626 | M2 vs M3: p-value = 1 |
| M4: Cubic spline for age | 4382.815 | M3 vs M4: p-value = 0.094 |
