# Supplementary material for: Development and validation of a model to predict ceiling of care in COVID-19 hospitalized patients
Source: BMC Palliat Care. 2024 Jul 16;23:173. doi: 10.1186/s12904-024-01490-8 (PMC11250965; doi:10.1186/s12904-024-01490-8)

**SUPPLEMENTARY FILE 4**

Figure 1: Receiver operating characteristic (ROC) curve, Area under the ROC curve and Brier Score for COPD


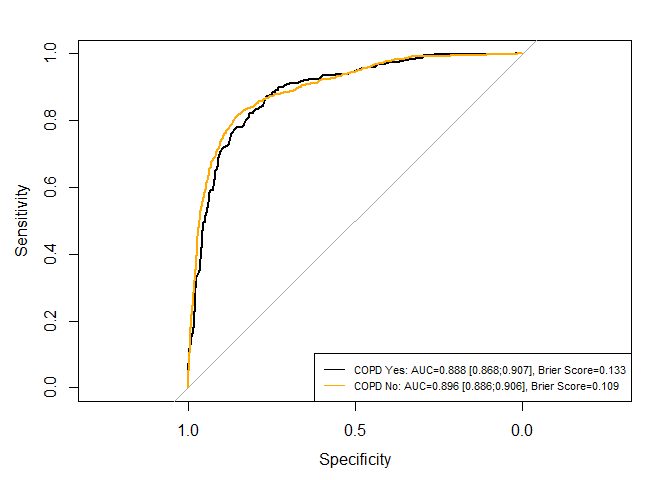


Figure 2: Observed vs predicted risk of the ceiling of care in the development cohort in patients with COPD


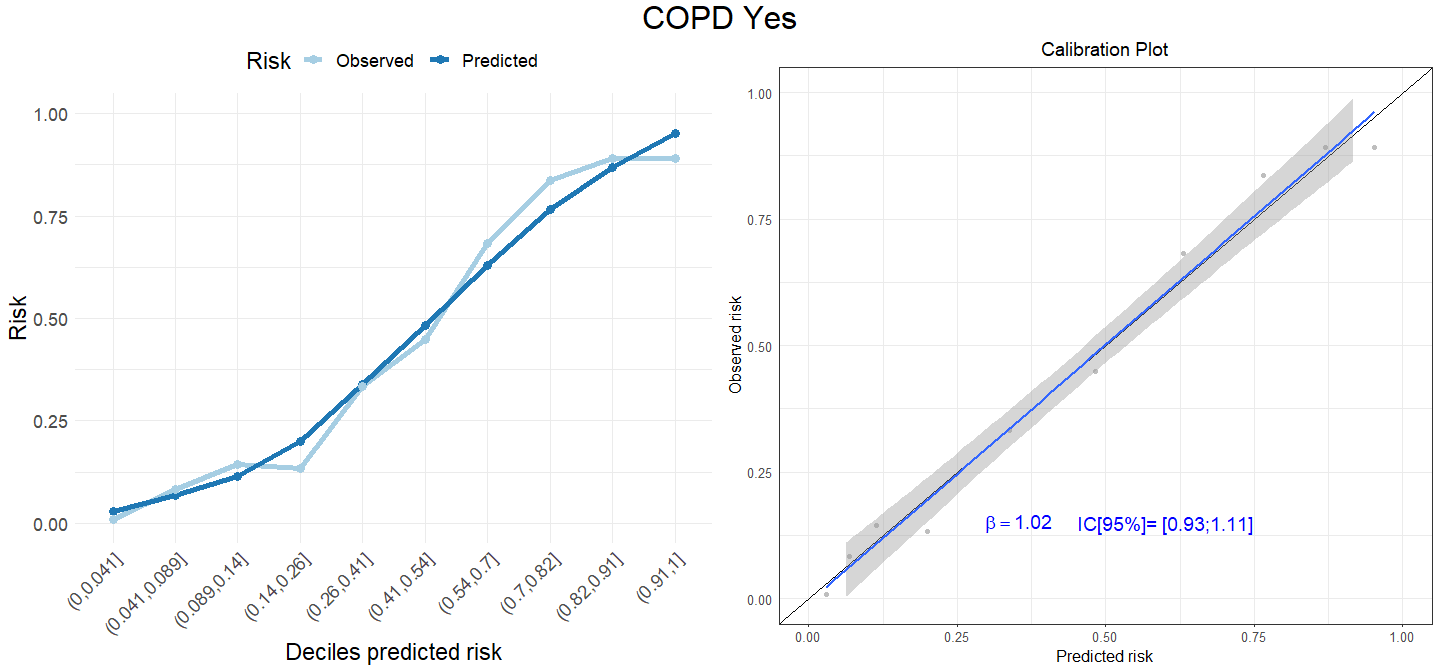


Figure 3: Observed vs predicted risk of the ceiling of care in the development cohort in patients without COPD


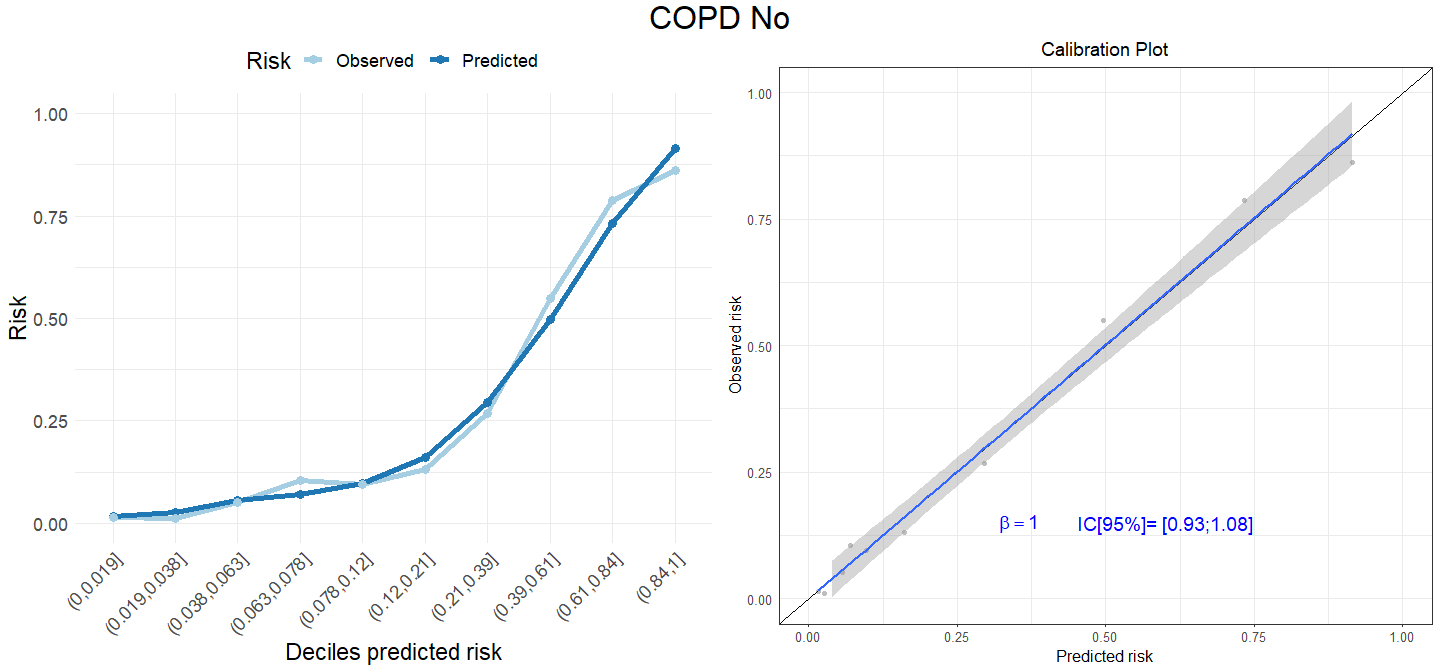


Figure 4: Receiver operating characteristic (ROC) curve, Area under the ROC curve and Brier Score for hypertension
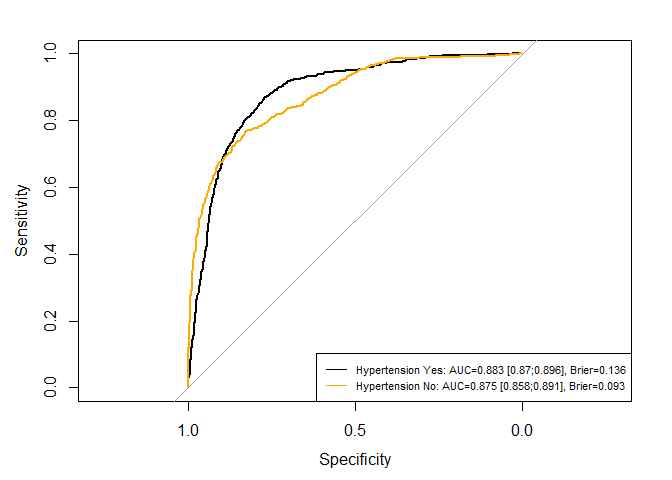


Figure 5: Observed vs predicted risk of the ceiling of care in the development cohort in patients with hypertension


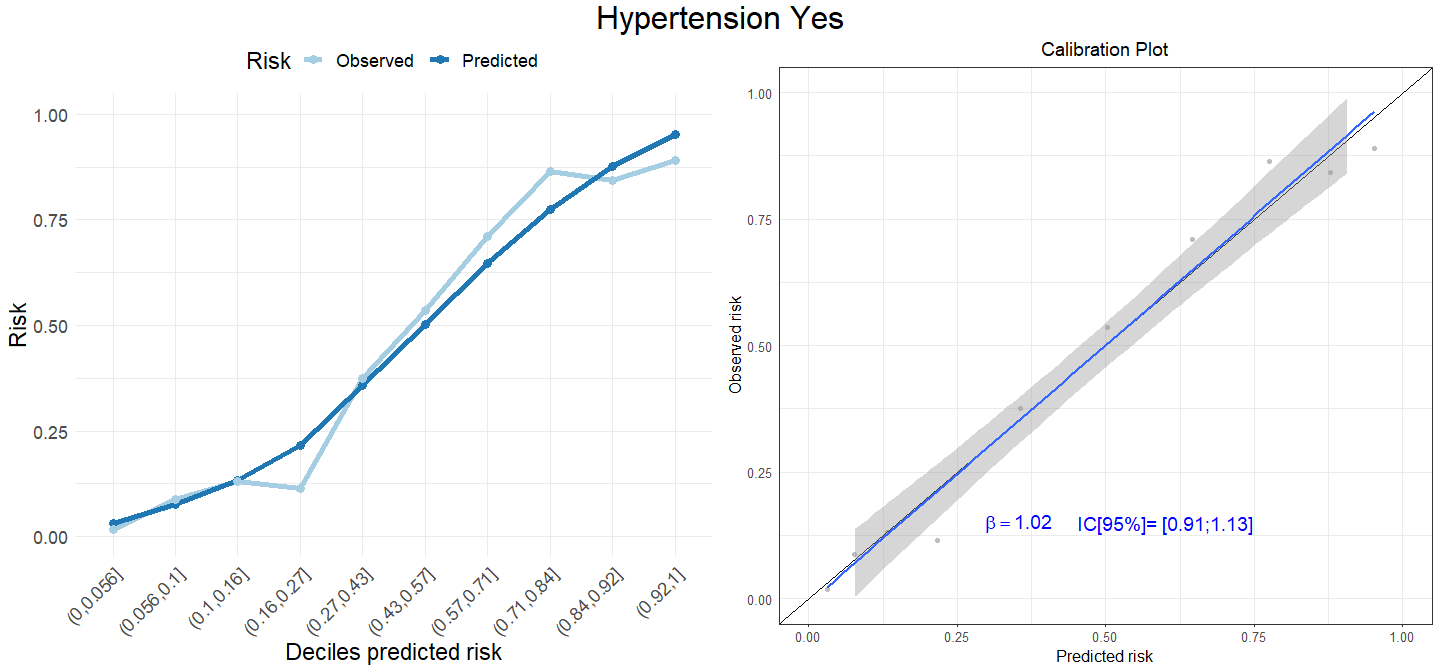


Figure 6: Observed vs predicted risk of the ceiling of care in the development cohort in patients without hypertension


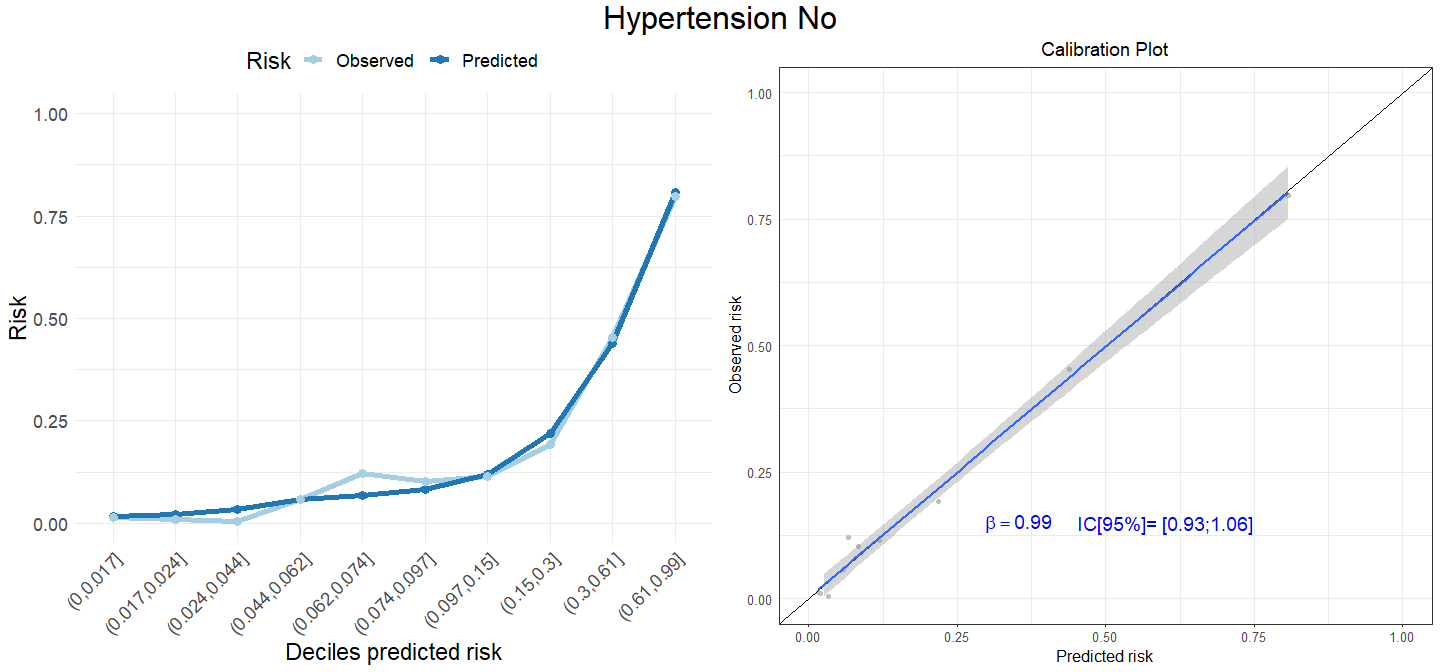


Figure 7: Receiver operating characteristic (ROC) curve, Area under the ROC curve and Brier Score for diabetes mellitus


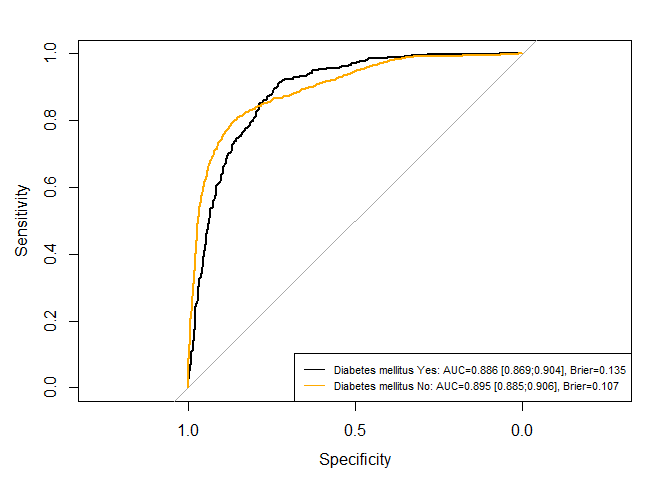


Figure 8: Observed vs predicted risk of the ceiling of care in the development cohort in patients with diabetes mellitus


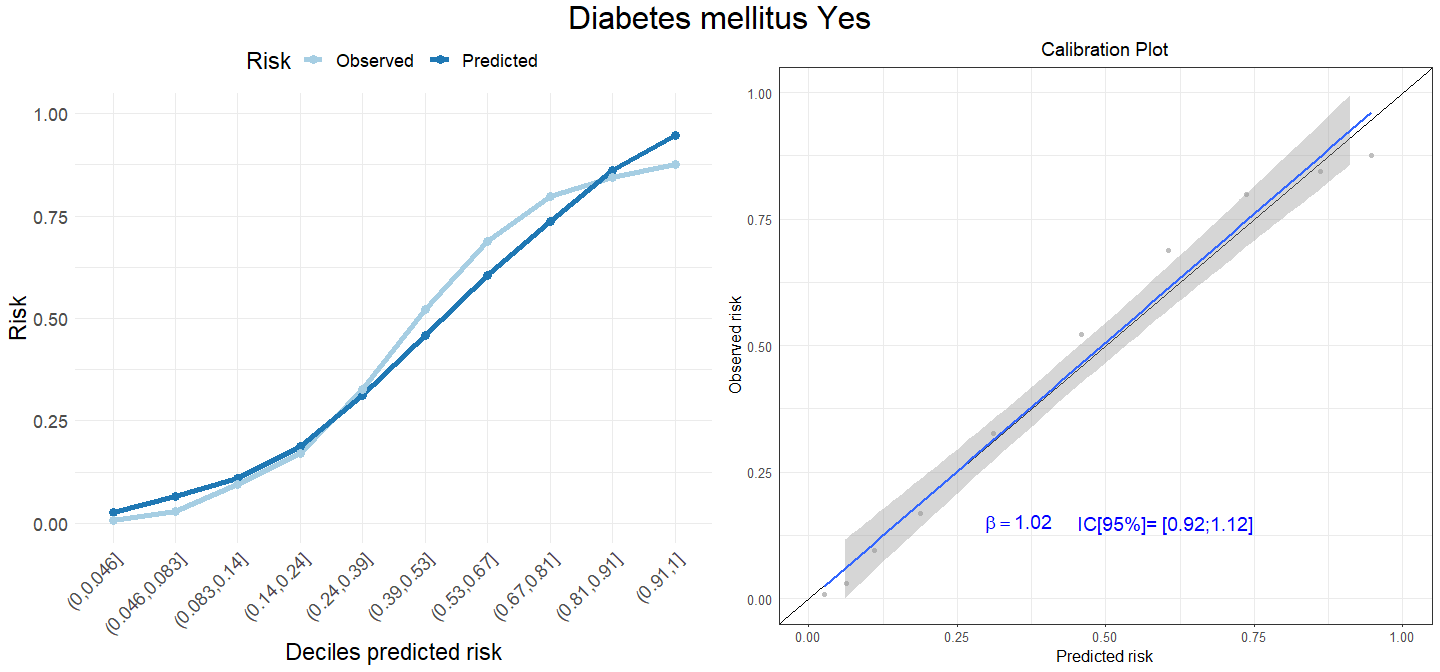


Figure 9: Observed vs predicted risk of the ceiling of care in the development cohort in patients without diabetes mellitus


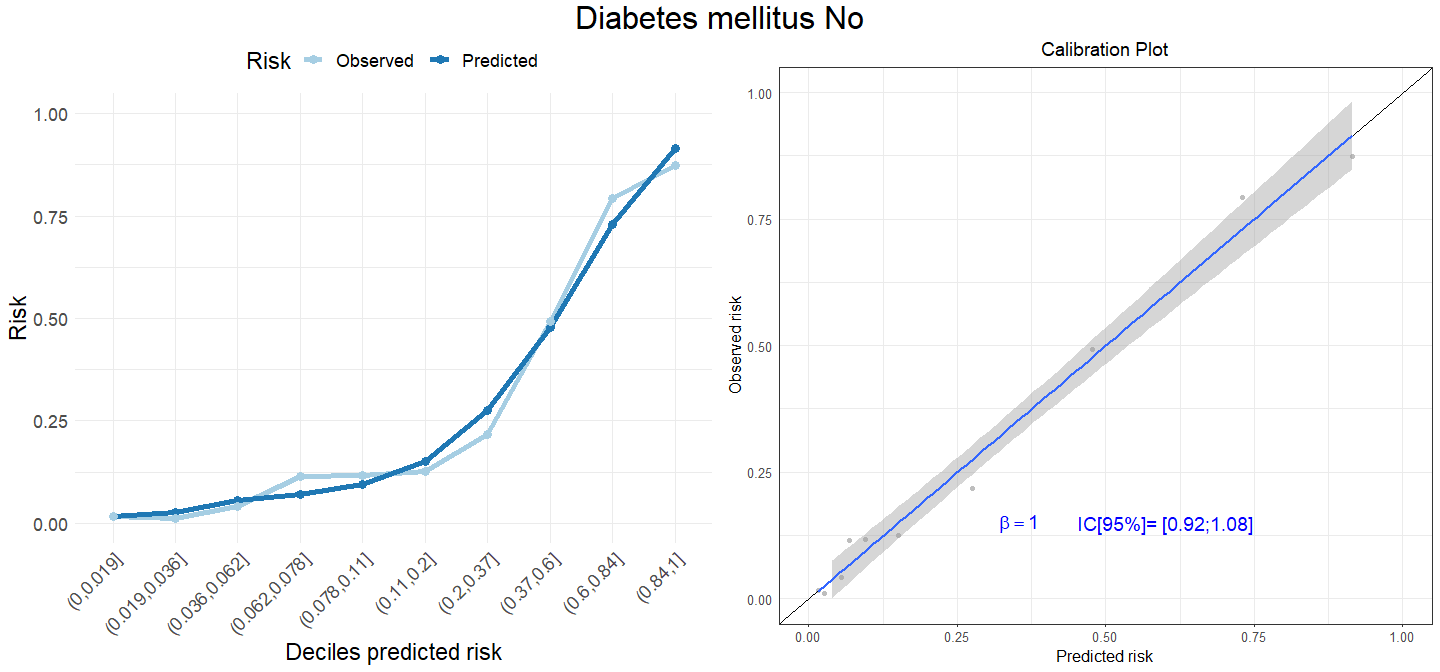


Figure 10: Receiver operating characteristic (ROC) curve, Area under the ROC curve and Brier Score for sex


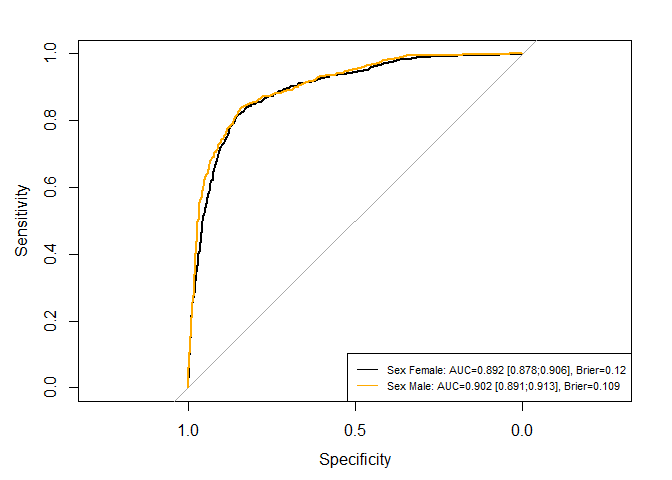


Figure 11: Observed vs predicted risk of the ceiling of care in the development cohort in female patients


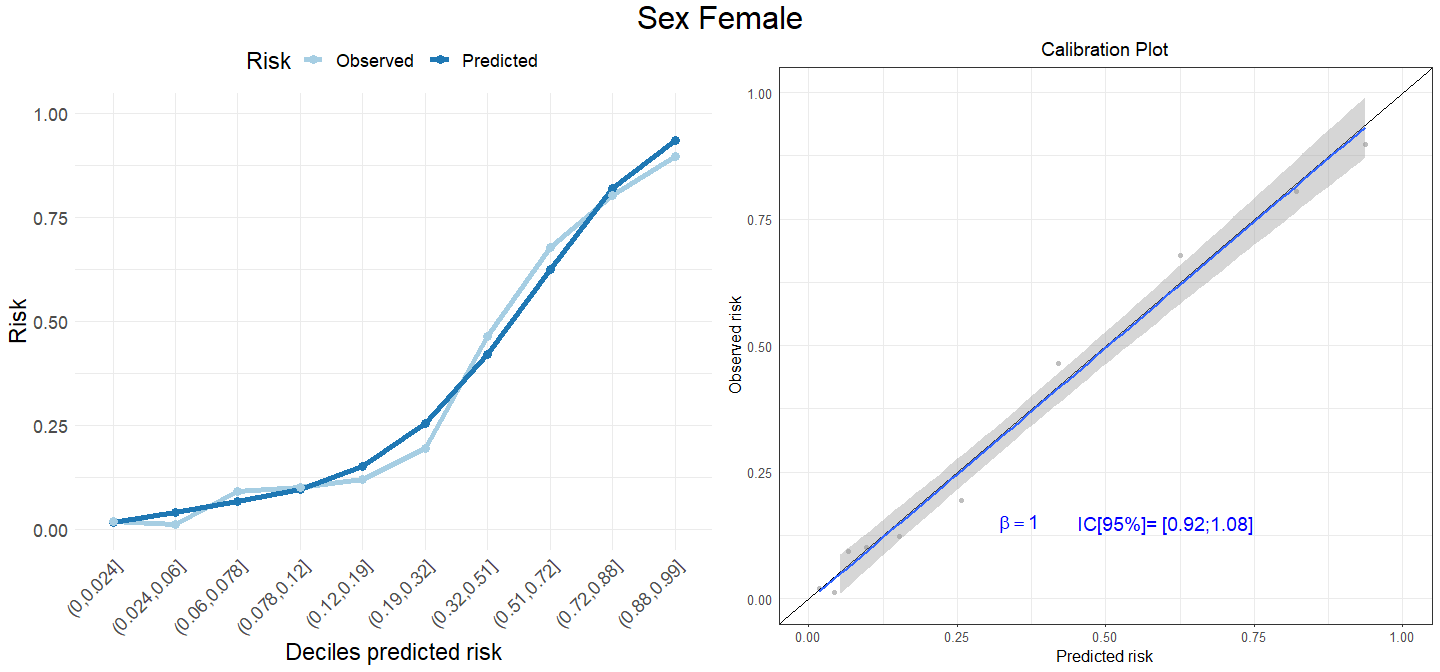


Figure 12: Observed vs predicted risk of the ceiling of care in the development cohort in male patients


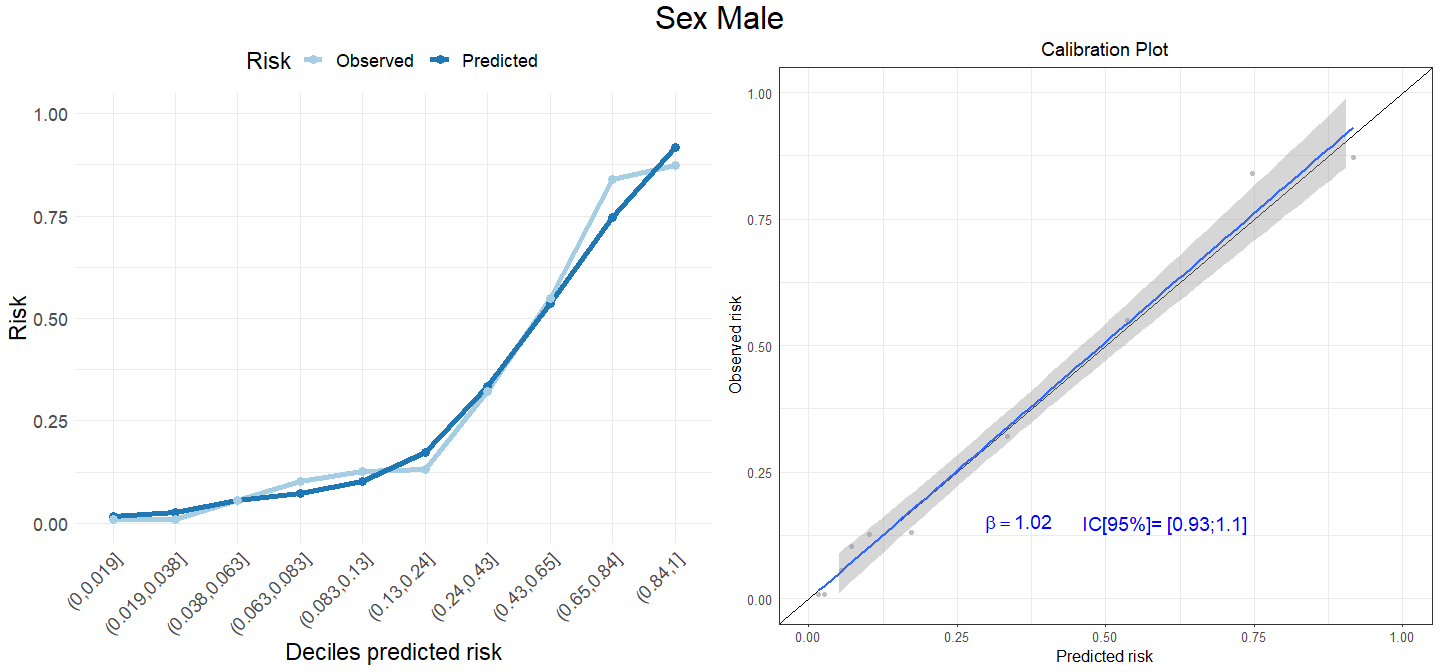


Figure 13: Receiver operating characteristic (ROC) curve, Area under the ROC curve and Brier Score for age


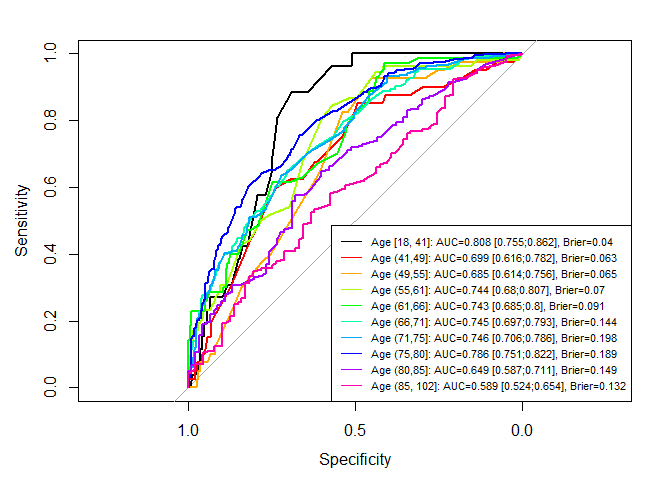


Figure 14: Observed vs predicted risk of the ceiling of care in the development cohort by deciles of age


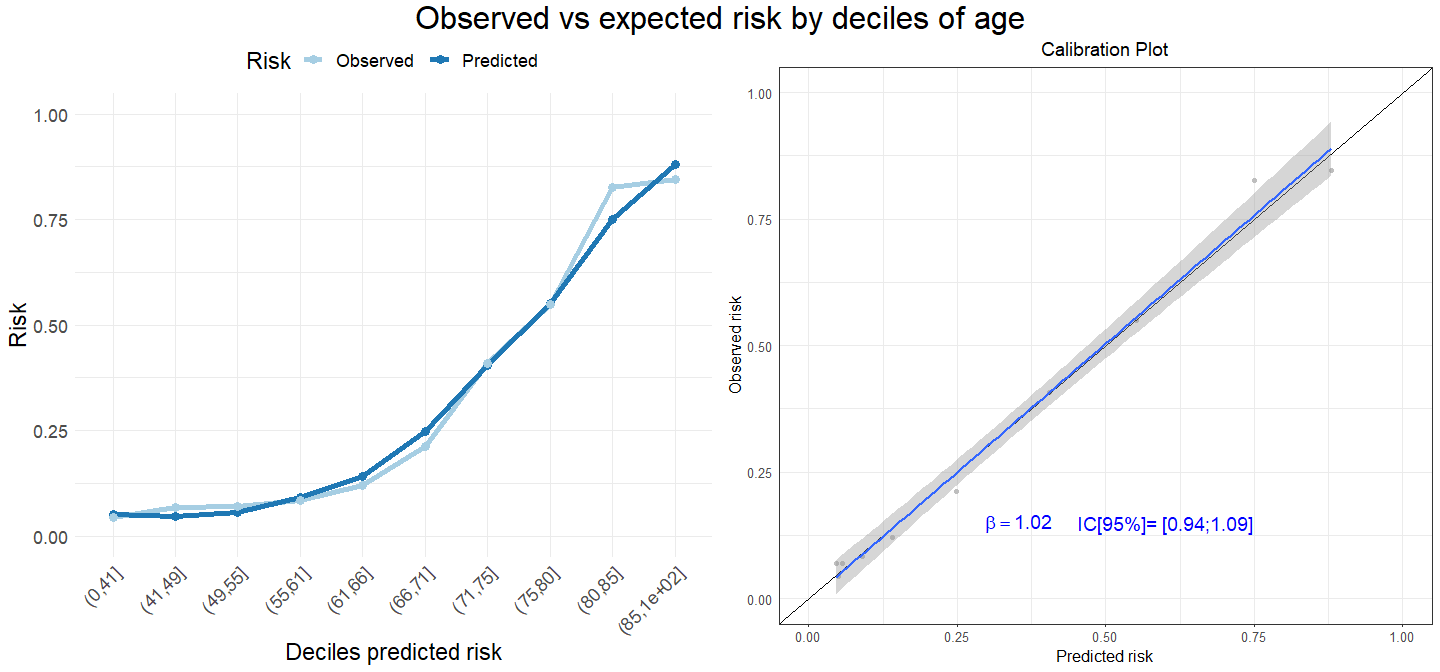

Supplement: Supplementary file 4 — Supplementary Material 4. [file 12904_2024_1490_MOESM4_ESM.docx]
